# Anterior horn damage in brachial multisegmental amyotrophy with superficial siderosis and dural tear: an autopsy case report

**DOI:** 10.1186/s12883-023-03180-z

**Published:** 2023-03-29

**Authors:** Yusuke Takahashi, Minori Kodaira, Mitsunori Yamada, Kai Uehara, Kiyoshi Ito, Tomoki Kaneko, Hiroki Ohashi, Masahide Yazaki, Yoshiki Sekijima

**Affiliations:** 1 Department of Medicine (Neurology and Rheumatology), Shinshu University School of Medicine, 3-1-1 Asahi, Matsumoto, 390-8621 Japan; 2 Department of Brain Disease Research, Shinshu University, Matsumoto, Japan; 3 Department of Laboratory Medicine, Shinshu University School of Medicine, Matsumoto, Japan; 4 Department of Neurosurgery, Shinshu University School of Medicine, Matsumoto, Japan; 5 Department of Radiology, Shinshu University School of Medicine, Matsumoto, Japan; 6 Department of Neurosurgery, Jikei University School of Medicine, Tokyo, Japan; 7 Institute for Biomedical Sciences, Shinshu University, Matsumoto, Japan; 8 Department of Clinical Laboratory Sciences, Shinshu University School of Health Sciences, Matsumoto, Japan

**Keywords:** Superficial siderosis, Brachial multisegmental amyotrophy, Dural tear, Snake-eyes appearance, Anterior horn

## Abstract

**Background:**

Patients with superficial siderosis (SS) rarely show brachial multisegmental amyotrophy with ventral intraspinal fluid collection accompanied with dural tear.

**Case presentation:**

We describe spinal cord pathology of a 58-year-old man who developed brachial multisegmental amyotrophy with ventral intraspinal fluid collection from the cervical to lumbar spinal levels accompanied with SS, dural tear, and snake-eyes appearance on magnetic resonance imaging (MRI). Radiological and pathological analyses detected diffuse and prominent superficial deposition of hemosiderin in the central nervous system. Snake-eyes appearance on MRI expanded from the C3 to C7 spinal levels without apparent cervical canal stenosis. Pathologically, severe neuronal loss at both anterior horns and intermediate zone was expanded from the upper cervical (C3) to middle thoracic (Th5) spinal gray matter, and these findings were similar to compressive myelopathy.

**Conclusion:**

Extensive damage of the anterior horns in our patient may be due to dynamic compression induced by ventral intraspinal fluid collection.

## Background

Superficial siderosis (SS) is a disease characterized by hemosiderin deposition on the surface of the central nervous system (CNS) due to chronic bleeding in the subarachnoid space [1, 2]. Patients with SS usually show slow progressive cerebellar ataxia, deafness, spasticity, and/or dementia [1]. Various disorders such as trauma, arteriovenous malformation, amyloid angiopathy, tumor, and surgery of the CNS can induce SS. However, recent studies have demonstrated that the most common etiology of SS is dural tear at the lower cervical and thoracic spine with or without trauma (i.e., duropathies) [2, 3]. A small proportion of these patients with ventral intraspinal fluid collection develop brachial multisegmental amyotrophy [4–6]. T2-weighted magnetic resonance imaging (MRI) in some patients with brachial multisegmental amyotrophy shows bilateral hyperintensity at the anterior horn of the cervical spinal cord, which produces a so-called “snake eyes” appearance in the images [5, 6]. However, pathophysiology of brachial multisegmental amyotrophy with ventral intraspinal fluid collection has not been elucidated. Herein, for the first time, we describe spinal cord pathology in an autopsied patient with brachial multisegmental amyotrophy accompanied with SS, dural tear, and snake-eyes appearance.

## Case presentation

A man without any family history of neurological diseases had four traffic accidents at the ages of 13, 19, 35, and 39 years. The traffic accidents did not induce or exacerbate any neurological symptoms. At the age of 38 years, he gradually developed progressive muscle atrophy and weakness in the predominantly proximal bilateral upper limbs. He showed tinnitus and hearing impairment at the age of 50 years. At the age of 53 years, brain and spinal cord MRI detected extensive SS. He was transferred to our hospital.

**Fig. 1:**
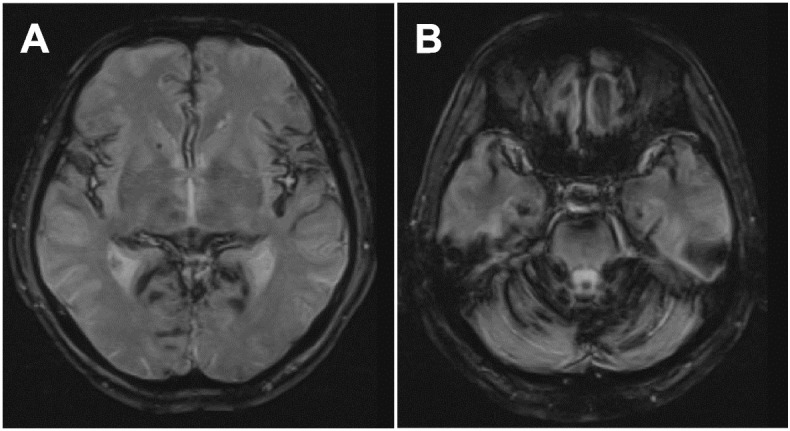
Brain MRI findings. Diffuse and prominent superficial deposition of hemosiderin in the brain on T2*-weighted imaging (**A**,** B**)

**Fig. 2:**
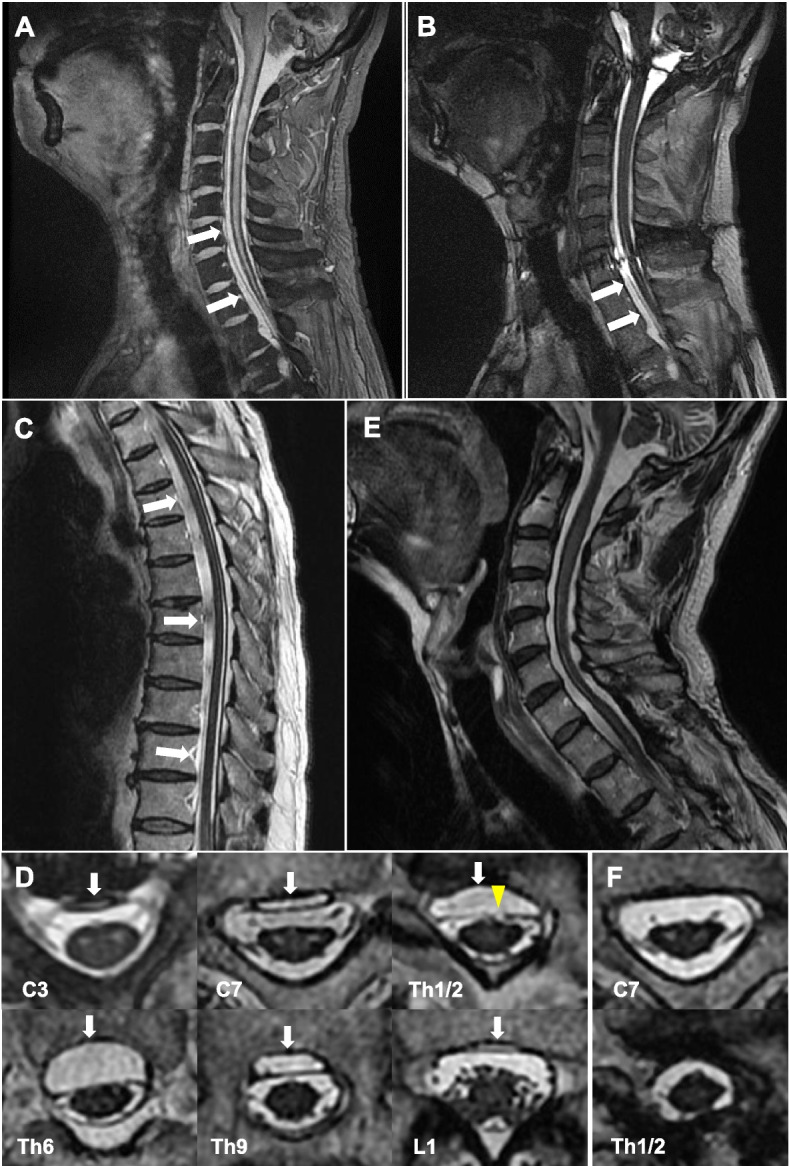
Spinal cord MRI findings. Superficial deposition of hemosiderin in the cervical cord (**A**). Ventral intraspinal fluid collection ranging from C3 to L1 spinal levels indicated by arrow (sagittal: **A**,** B**,** C**; axial: **D**). Snake-eyes appearance from the C3 to C7 spinal levels (**D**). Discontinuous part of the dura at the Th1/2 spinal level (arrowhead) (**D**). Postoperative remnant snake-eyes appearance without recurrence of ventral intraspinal fluid collection (sagital: **E**; axial: **F**). **A**: T2*-weighted imaging, **B-F** T2-weighted imaging

Upon admission, mental and cognitive state were normal. Except for moderate hearing loss, cranial nerve functions were preserved. Bilateral muscle atrophy was apparent in the predominantly proximal upper limbs. He showed upper limb weakness (i.e., supraspinatus, 3/3 (right/left); pectoralis major 3/3; deltoid, 2/3; biceps, 3/4; triceps, 4/4; wrist extensor, 5/4; wrist flexor, 5/5; finger extensor, 5/2; dorsal interossei, 3/4 on the Medical Research Council Scale (MRC, 0–5)), without lower limb weakness. There were no sensory or autonomic dysfunctions. Tendon reflex in the upper limbs was decreased or diminished. Tendon reflex in the lower limbs was brisk without any pathological reflexes. He showed wide-based gait and was not able to perform tandem gait. No abnormal blood test findings were detected. Appearance of the cerebrospinal fluid (CSF) was bloody. Motor nerve conduction studies (NCSs) of the right limbs detected decreased amplitude of compound muscle action potentials in the median (780 μV) and ulnar (4.5 mV) nerves. F-wave occurrence of the median nerve was also decreased (30%). However, delay of F-wave and distal latency was mild, and motor nerve conduction velocity was preserved. Results of sensory NCSs in the upper limbs and those of motor and sensory NCSs in the lower extremities were normal. On electromyography (EMG) of the right extremities, apparent chronic denervation and reinnervation changes with scarce active denervation changes were detected in the upper limbs. In the quadriceps femoris muscle, EMG findings were normal. Transcranial magnetic stimulation showed prolonged central conduction time in the upper and lower limbs. T2*-weighted imaging of the brain (Fig. 1) and spinal (Fig. 2A) MRI detected extensive SS. Ventral intraspinal fluid collection ranging from the C3 to L1 spinal levels was also detected (Fig. 2A-D, indicated by arrow). T2-weighted imaging showed high-intensity lesions at the bilateral anterior horn with snake-eyes appearance, ranging from the C3 to C7 spinal levels without apparent cervical canal stenosis (Fig. 2B, D). MRI also revealed a discontinuous part of the dura at the Th1/2 spinal level, suggesting dural tear (Fig. 2D, indicated by arrow head). We diagnosed him with brachial multisegmental amyotrophy accompanied with SS and dural tear. At the age of 54, repair of dural tear at the spinal level of Th2 were performed. Although temporary deterioration of ataxia occurred following the operation, progression of neurological dysfunctions stopped thereafter. At the age of 56, clear appearance of the CSF was confirmed by lumbar puncture. The snake-eyes appearance remained in MR images without recurrence of ventral intraspinal fluid collection (Fig. 2E, F). At the age of 58 years, he accidentally died due to drowning in the bath. Autopsy was performed with permission from his family.

**Fig. 3:**
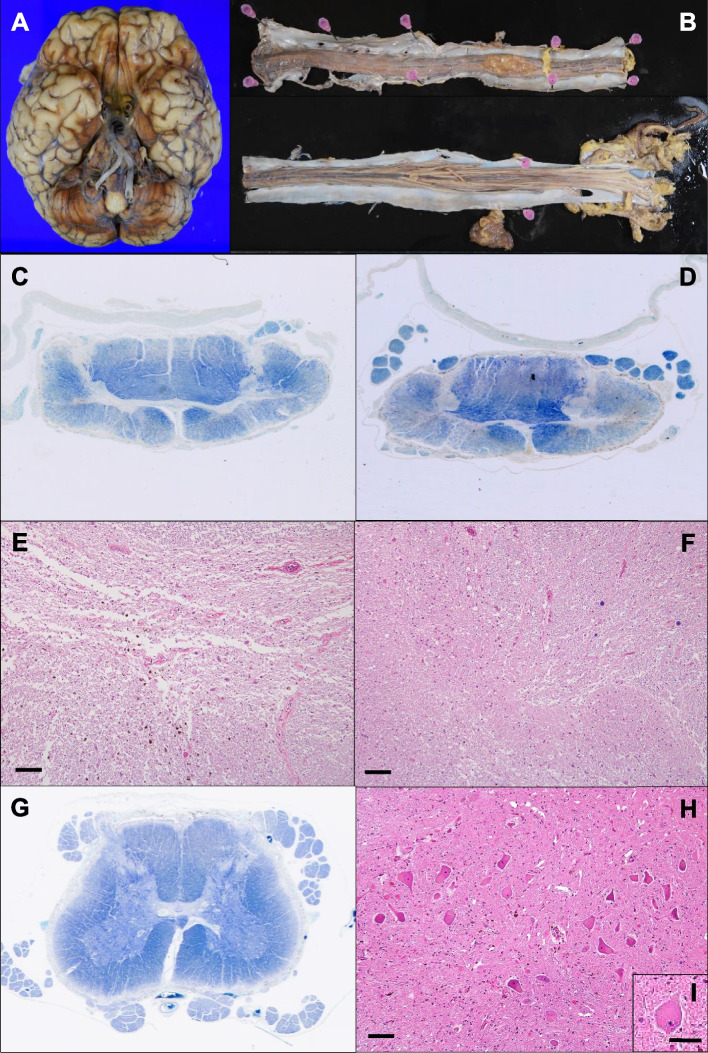
Pathological findings. Extensive hemosiderin deposition on the brain (**A**) and spinal cord (**B**). Marked atrophy of the spinal gray matter such as the anterior horns and intermediate zone at the C5 (**C**) and C8 (**D**) segments. Severe neuronal cell loss without apparent chromatolysis in both anterior horns and intermediate zone at the C5 segment (**E**). No obvious tissue damage in the posterior horns at the C5 segment (**F**). Relatively preserved spinal gray matter at the L4 segment (**G**). Persistence of many motor neurons in the anterior horn at L4 (**H**). A chromatolytic neuron in Fig. 3H (**I**). **C**,** D**,** G** Klüver-Barrera staining, **E**,** F**,** H, I** Hematoxylin and eosin staining. Scale bars in **E**,** F**,** H** are 100 μm. Scale bar in **I** is 50 μm

Macroscopically, the brain and spinal cord were brown due to extensive hemosiderin deposits (Fig. 3A, B). Microscopically, the cerebellum showed severe loss of the Purkinje cells along with hemosiderin deposition. In the spinal cord, hemosiderin deposition was nearly even throughout the surface; however, severe tissue damage of the spinal gray matter such as the anterior horns and intermediate zone was observed from the C3 to Th5 segments (Fig. 3C-F). The damage was more accentuated in segments from the C5 to Th2. In the anterior horns at these segments, neuronal cells were severely depleted, and remaining neurons were atrophic (Fig. 3E). Chromatolysis, known as axonal reaction, was not apparent in the remaining neurons. In contrast to the anterior horns and intermediate zone, no obvious tissue damage was observed in the posterior horns (Fig. 3F). Anterior horn cells were relatively preserved in segments from the middle thoracic (Th7) to lumbar cord (Fig. 3G, H); however, a small amount of these neurons showed chromatolysis (Fig. 3I). Mild degeneration of the white matter due to superficial hemosiderin deposition was also observed in the superficial region of the entire spinal cord (Fig. 3C, D, G).

## Discussion

Brachial multisegmental amyotrophy is a very rare phenotype among patients with SS and ventral intraspinal fluid collection [2]. In previous clinical and radiological case studies [4–6], two speculative theories for motor dysfunction in patients with brachial multisegmental amyotrophy have been propsed; 1) compressive damage to the anterior horns from ventral intraspinal fluid collection, and 2) stretching damage to the motor nerve roots induced by posterior shift of the spinal cord due to ventral intraspinal fluid collection [4–6].

In our patient, snake-eyes appearance was detected on cervical MRI, similar to that in some of reported cases of brachial multisegmental amyotrophy with SS and dural tear [5, 6]. Snake-eyes appearance on cervical spinal cord MRI had been described in patients with brachial mutisegmental amyotrophy caused by cervical spondylosis or ossification of the posterior longitudinal ligament (OPLL) [7, 8]. To our knowledge, patients with snake-eyes appearance who have sole damage to the nerves such as neuropathy, radiculopathy, and plexopathy have not been described previously. Pathological analysis of an autopsied patient with OPLL and snake-eyes appearance on MRI has shown intramedullary cystic necrosis around the central gray matter and the ventolateral posterior column, with loss of the anterior horn cells [8]. Autopsied case series of cervical spondylotic myelopathy have demonstrated similar neuropathological findings, and atrophy and neural loss are considered to start at the anterior horns and intermediate zone of the spinal gray matter [9]. Pathophysiology of compressive spinal cord damage has been postulated to be due to circulatory disturbance [7–9]. In our patient, snake-eyes appearance on MRI and distribution of spinal cord damage on pathological analysis were similar to those findings in patients with compressive myelopathy [8, 9]. The similarity of histological changes of the spinal gray matter between spondylotic myelopathy and the present case suggests the pivotal role of ventral intraspinal fluid collection in the development of the anterior horn damage.

On the other hand, amount of ventral intraspinal fluid collection was too small to severely compress the cervical spinal cord on MRI. Compared with the cervical spinal cord level, the amount of ventral intraspinal fluid collection was large at the thoracic spinal cord level especially in the middle thoracic spine. Reasons for the discrepancy in the levels between conspicuous anterior horn cell loss at the middle cervical to upper thoracic cord and the largest amount of ventral intraspinal fluid collection at the middle thoracic cord are unclear. However, a larger range of motion in the cervical spine than in the thoracic spine could be related to this discrepancy. Indeed, we assessed the spinal cord on MRI in a neutral position alone. In Hirayama disease, MRI at a neck flexion position can detect compression of the cervical spinal cord induced by forward displacement of the cervical dural sac [10]. Cervical MRI during neck extension and/or flexion might show dynamic compression of the cervical cord due to ventral intraspinal fluid collection. Further studies are needed on the dynamic change of ventral intraspinal fluid collection and spinal cord following posture change.

As for the idea that ventral intraspinal fluid collection may stretch the motor nerve roots causing brachial multisegmental amyotrophy, extensive damage of the anterior horns and intermediate zone with snake-eyes appearance on MRI in our patient is unlikely to be caused by damage to the motor nerve roots alone.

Diffuse superficial deposition of hemosiderin on the whole segment of the spinal cord on our pathological analysis does not support that brachial multisegmental amyotrophy is mainly induced by hemosiderin deposition. However, a small amount of motor neurons with chromatolysis were detected in the segment with preserved anterior horn cells, and this finding may be due to damage to the axons of motor neurons in the anterior horns induced by hemosiderin deposition. No apparent chromatolysis at the segments with severe anterior horn damage may be due to depletion of almost all large neurons corresponding to motor neurons at these segments. On the other hand, some patients with both brachial multisegmental amyotrophy and ventral intraspinal fluid collection do not show SS on brain MRI [5]. Furthermore, Morishima et al., reported a case with neither SS on brain MRI nor red blood cells in the CSF [5]. These reports also suggest that hemosiderin deposition is not essential for developing brachial multisegmental amyotrophy in patients with ventral intraspinal fluid collection. Further studies are needed to elucidate the pathophysiology of brachial multisegmental amyotrophy accompanied with SS and dural tear.

## Conclusions

We pathologically confirmed selective neuronal loss of both anterior horns and intermediate zone at the upper cervical to middle thoracic spinal gray matter in a patient with brachial multisegmental amyotrophy accompanied with SS, dural tear, and snake-eyes appearance on MRI. Extensive anterior horn damage in our patient may be due to dynamic compression induced by ventral intraspinal fluid collection.

## Data Availability

All data analysed are included in this published article.

## Abbreviations

SS: Superficial siderosis
MRI: Magnetic resonance imaging
NCSs: Nerve conduction studies
EMG: Electromyography
OPLL: Ossification of the posterior longitudinal ligament
